# Molecular analysis of *BRCA1* and *BRCA2* genes in La Rioja (Spain): five new variants

**DOI:** 10.1186/s13053-024-00296-2

**Published:** 2024-10-22

**Authors:** Raquel Salazar Saez, Miriam Zorrilla, Rosa Sánchez, Ana Cebollero, Isabel Manrique, Alfonso Martín, Leticia de Ávila, Alejandra Lacalle-Emborujo, Samuel Martin-Rodriguez, Iván Bernardo-González, Martina Alonso

**Affiliations:** 1 Clinical Oncology Unit, Hospital Universitario San Pedro de Logroño, La Rioja, Spain; 2 Biomedical Diagnosis, Hospital Universitario San Pedro de Logroño, La Rioja, Spain; 3 Análisis Clínicos, Hospital Universitario San Pedro de Logroño, La Rioja, Spain; 4 Servicio de Oncología Médica, Hospital Universitario San Pedro, 3ª planta, Calle Piqueras nº 98, Logroño, La Rioja, 26006 Spain

**Keywords:** *BRCA1*, *BRCA2*, Breast and ovarian cancer, Hereditary cancer, Pathogenic variants, La Rioja population

## Abstract

**Background:**

To study *BRCA1/2* gene variants in La Rioja in the northcentral area of Spain.

**Methods:**

We performed a molecular analysis of *BRCA1* and *BRCA2* in 642 individuals from 427 different families from June 2008 to December 2019.

**Results:**

We identified 71 families with pathogenic variants in these genes, 32 families with *BRCA1* variants and 39 families with *BRCA2* variants. The pathogenic variants c.959delG in *BRCA1* and c.1363_1369delTCAGAGA, c.1397dupA, c.4234_4236delACTinsC and c.8387delC in *BRCA2* have not been previously described. The c.81–2 A > T variant in *BRCA1*, detected in two unrelated families, has not been reported previously in the Spanish population. Two large genomic deletions were found in the *BRCA1* gene in exons (Ex) 23–24 and Ex1A-1B-2, and one deletion was found in the *BRCA2* gene in Ex2. The pathogenic variant c.5123 C > A in *BRCA1* was detected in 8 unrelated families and was the most frequent pathogenic variant in our population. The c.6024dupG mutation in *BRCA2* was detected in 6 unrelated families; the c.2808_2011delACAA mutation in *BRCA2* was found in 5 different families; the c.211 A > G mutation in *BRCA1* was found in three different families; and the c.68_69delAG, c81-2 A > T, c.4038_4039delAA, and c.5266dupC variants in *BRCA1* and the c.2457delA, c.2701delC, c.5116_5119delAATA, c.6275delTT, c.7558 C > T and c.7617 + 1G > A variants in *BRCA2* were found in two different families.

**Conclusions:**

The spectrum of pathogenic variants in the *BRCA1/2* genes in La Rioja is similar to that in other Spanish regions, with some unique characteristics. The pathogenic c.6024dupG variant in the *BRCA2* gene was detected in a large number of families and could have a founding effect in the Ebro riverside areas in the regions of La Rioja and Navarra.

**Trial registration:**

Not applicable.

**Supplementary Information:**

The online version contains supplementary material available at 10.1186/s13053-024-00296-2.

## Background

Familial susceptibility to breast cancer accounts for < 25% of all breast cancer cases. *BRCA1* and *BRCA2* are high-penetrance breast cancer predisposition genes that were identified in the 1990s by genome-wide linkage analysis and positional cloning. Pathogenic variants in *BRCA1/2* explain ∼20% of familial clustering of breast cancer cases. The estimated population frequency of pathogenic variants in *BRCA1/2* genes is 1/800–1/1000 per gene [1]. The prevalence of germline pathogenic *BRCA1* or *BRCA2* variants varies considerably among ethnic groups and geographical areas [1].

The identification of pathogenic variants in *BRCA1* or *BRCA2* has important clinical implications, as the presence of these variants is an important factor in risk assessment and medical management of patients. Interventions, such as annual screening by breast magnetic resonance imaging (MRI), are available for women harboring deleterious *BRCA1* or *BRCA2* variants to facilitate early detection of breast cancer and active risk reduction via risk-reducing surgery, such as bilateral mastectomy and salpingo-oophorectomy [2]. The presence of pathogenic *BRCA1* or *BRCA2* variants may also influence cancer treatment decisions, mainly regarding the use of platinum agents, poly (ADP-ribose) polymerase (PARP) inhibitors [3] or contralateral risk-reducing mastectomy.

Increasing numbers of women are undergoing clinical genetic testing for *BRCA1* and *BRCA2* pathogenic variants, and recommendations for the patients to whom testing should be offered continue to expand [4].

Spain is divided into 17 autonomous communities. Each community was composed of one or more provinces. The community of La Rioja has only one province, and by 2023, it had a population of 324,226 inhabitants (Instituto Nacional de Estadística. National Statistics Institute). Other autonomous communities had previously carried out genetic studies of BRCA1/2 [7–10], but La Rioja did not carry out any studies. In 2008 we began to study this population. In this study, the pathogenic variants found in the population of this Spanish region are presented for the first time.

## Methods

### Study population

Families were collected from June 2008 to December 2019 and selected by the Cancer Genetic Counselling Unit according to the criteria of the Spanish Society of Medical Oncology (SEOM), as detailed in Table 1.

**Table 1:** Genetic study criteria

Study Criteria	Clinical Pathological Features
GROUP I 1 case of cancer in the family	A. Female with breast cancer and synchronous or metachronous ovarian cancer B. Breast cancer ≤ 35 years old (or ≤ 40 years old in case of noninformative family^a^) C. Bilateral breast cancer (first diagnosed ≤ 40 years old) D. Triple-negative breast cancer ≤ 50 years old E. High-grade serous ovarian cancer (or fallopian tube or primary peritoneal)
GROUP II 2 cases of cancer in the family^b^	A. Bilateral breast cancer + other breast cancer ≤ 50 years old B. Male breast cancer + breast or ovarian cancer in females C. Breast cancer + ovarian cancer D. Two cases of breast cancer diagnosed ≤ 50 years old
GROUP III 3 cases of cancer in the family^b^	≥ 3 cases of breast cancer ± ovarian cancer

^a^ Fewer than 2 females who lived to age 45 years on each side of the family. ^b^ On the same side of the family

The clinical criteria for *BRCA1-2* genetic testing are based on personal and family histories of cancer, with the aim of achieving a detection rate of at least 10% of pathogenic variants. This criterion should not be considered when the objective of a genetic study is to identify whether breast or ovarian cancer patients harboring a pathogenic variant in a gene associated with a therapeutic target can benefit from targeted therapy. These patients were included in groups I-D and I-E.

Information collected from each family included the type of cancer, age at diagnosis, age at death, current age, and geographic origin and was used to generate a pedigree. Genetic testing was offered to affected individuals from high-risk families or to healthy relatives from families in which a pathogenic variant had been detected. All tested individuals signed an informed consent form after appropriate genetic counseling. More than 99% of the individuals referred for genetic counseling agreed to participate in the genetic study. Blood samples were obtained via peripheral venous extraction at San Pedro Hospital or at the corresponding health center. The study was approved by the hospital´s ethics committee.

### Pathogenic variant analysis

Venous blood samples were collected in EDTA tubes, and genomic DNA was extracted from peripheral blood leukocytes following a standard protocol with a MagNA Pure Compact Nucleic Acid Isolation Kit (Roche, Basel, Switzerland).

### Sanger sequencing

Initially, polymerase chain reaction (PCR) amplification was performed with primers obtained from the literature [5]. Subsequently, PCR amplification was performed with EasySeq^™^ PCR plates for *BRCA1/2* sequencing. In both cases, the amplicons obtained were subsequently sequenced using the BigDye Terminator v1.1 Cyclic Sequencing Kit (Applied Biosystems, Forrest City, CA) and analyzed on an ABI 3130 automated sequencer (Applied Biosystems). Sample reads were compared with the reference gene sequence (GRCh37/hg19 assembly) using SeqScape v2.5 software.

Multiplex ligation-dependent probe amplification (MLPA) analyses were performed using the SALSA MLPA Probemixes P002 *BRCA1*, P087 *BRCA1* Confirmation, P090 *BRCA2*, P003 *MLH1*/*MSH2* and P043 *APC* from MRC Holland (Amsterdam).

### Next-generation sequencing

We initially used a custom targeted panel to capture the exons and flanking introns of 27 genes involved in hereditary cancers (March 2015). A SureSelect custom kit (Agilent, Santa Clara, CA) was used, and the libraries were run on a MiSeq sequencer (Illumina, San Diego, CA) following a paired-end protocol. In February 2017, we generated libraries using TrueSight^®^ Hereditary Cancer, a commercial panel of 94 cancer-related genes manufactured by Illumina, and ran them on a MiSeq or NexSeq sequencer. Sample reads were aligned to the reference human genome (GRCh37/hg19) using Burrows–Wheeler Aligner (BWA v0.7.15), and duplicate reads were marked using MarkDuplicates (Picard tools). Indel realignment, base quality recalibration, variant calling and variant filtering were performed using the Genome Analysis Toolkit (GATK v3.6). Finally, filtered variants were annotated using ANNOVAR [6].

Next-generation sequencing (NGS) studies have been carried out since March 2015, and slightly more than half of pathogenic variants have been detected by this method.

### Detection of copy number variants

The data obtained by NGS were used to detect copy number variants (CNVs) using a proprietary pipeline. Briefly, we sequenced 15 controls (the remaining patients in the series) via the same procedure as that used for our patient. For each sample, the reads were mapped to the reference human genome, and duplicate reads were flagged. We performed a coverage analysis using Picard’s CollectHsMetrics tool with the PER_TARGET_COVERAGE option. This tool provides sequence depth information, including normalized within-sample coverage for each target region (hereafter referred to as “ISNC”). Then, for each sample, a copy number ratio (CNR) was calculated for each target region by dividing the ISNC of the sample by the average ISNC of all samples (normalization across samples). The cutoff values have been previously validated with known CNV samples [5].

### Databases and tools used for variant analysis

- Pathogenicity databases: ClinVar, Biobase HGMD Professional.
- Population frequency databases: 1000 Genomes, ExAC, ESP6500.
- Generic databases: dbSNP, UniProt.
- In silico predictors: SIFT, PolyPhen, MutationTaster, CADD, Human Splicing Finder.

In the Genetics Laboratory of San Pedro Hospital in Logroño (Spain), a complete molecular study was carried out in 56 families. In the remaining 15 families, the pathogenic variants detected in other laboratories in neighboring autonomous communities in relatives residing in La Rioja were studied. Pathogenic variants were detected by Sanger sequencing in 36 patients, MLPA in 3 patients, and NGS in 32 patients.

## Results

Molecular analysis of the *BRCA1* and *BRCA2* genes was carried out in 642 individuals belonging to 427 different families. Pathogenic variants in either the *BRCA1* or *BRCA2* gene were detected in 71 families, representing 16.5% of the study population. A total of 32 families had pathogenic variants in the *BRCA1* gene, and 39 families had pathogenic variants in the *BRCA2* gene. All the included families resided in the community of La Rioja in north-central Spain. According to the study criteria, 3 families belonged to Group I-A, 36 to Group I-B, 6 to Group I-C, 6 to Group I-D, 36 to Group I-E, 14 to Group II-A, 10 to Group II-B, 34 to Group II-C, 102 to Group II-D and 170 to Group III (Table 2).

**Table 2:** Distribution of families and pathogenic variants according to the study criteria

Study criteria	Number of families studied	Families with pathogenic variants	Percentage
GROUP I	A. 3	A. 0	A. 0%
	B. 36	B. 4	B. 11.1%
	C. 6	C. 0	C. 0%
	D. 6	D. 2	D. 33.33%
	E. 37	E. 4	E. 10.81%
GROUP II	A. 14	A. 1	A. 7.1%
	B. 10	B. 2	B. 20%
	C. 34	C. 6	C. 17.64%
	D. 102	D. 11	D. 10.7%
GROUP III	170	39	22.04%

Eight families had only one case of breast cancer in a male. The pathogenic variant c.6024dupG in the *BRCA2* gene was detected in one family, accounting for 12.5% of this subgroup. The pathogenic *BRCA2* variant c.4234_4236delACTinsC was detected in a family with pancreatic cancer and melanoma, with the proband being a female with pancreatic cancer. In this family (Family 64), there were no cases of breast or ovarian cancer.

A total of 290 (67.9%) families had only breast cancer, 97 (22.7%) had both breast and ovarian cancer, and 39 (9.1%) had only ovarian cancer. In 21 (4.9%) families, there was a male patient with breast cancer.

In 25 families, there were no living index patients with cancer; therefore, the healthy patients who attended the genetic counseling consultation were analyzed. We detected a pathogenic variant in five families, representing 20% of this subgroup.

Group III (3 cancer cases) had the highest number of pathogenic variants (22%). No pathogenic variants were found in Groups I-A (females with breast cancer and synchronous or metachronous ovarian cancer) or I-C (bilateral breast cancer, first diagnosed ≤ 40 years old), although these groups included few patients (3 and 6, respectively). In Group I-D (triple-negative breast cancer diagnosed ≤ 50 years old), we identified two females with pathogenic variants out of the six included patients (33%) (Table 2).

In Group I-B (breast cancer diagnosed ≤ 35 years old or ≤ 40 years old in the case of a noninformative family), pathogenic variants in the *BRCA1/2* genes were detected in 11.1% of patients, and in Group I-E (high-grade serous ovarian, fallopian tube or primary peritoneal cancer), they were detected in 10.81% of patients. In Group II (two cases of cancer in the family), Group II-B (male breast cancer + breast or ovarian cancer in females) had the highest percentage of pathogenic variants (20%), followed by Groups II-C (breast cancer + ovarian cancer; 17.64%), II-D (two cases of breast cancer diagnosed ≤ 50 years old; 10.7%) and II-A (bilateral breast cancer + other breast cancer diagnosed ≤ 50 years; 7.1%) (Table 2).

### Pathogenic variants of the BRCA1 gene

The detected pathogenic variants of the *BRCA1* gene are listed in Table 3. *BRCA1* families (*n* = 32) had a total of 19 different pathogenic variants, including 6 recurrent pathogenic variants and 13 unique pathogenic variants.

**Table 3:** Germline pathogenic variants of *BRCA1* in breast and ovarian cancer families in La Rioja

Exon	HGVS nomenclature at cDNA level		HGVS nomenclature at protein level		dbSNP	Number of families	References: articles published with the variant
2	c.68_69delAG		p.Glu23ValfsTer17		rs80357914	2	[23–25]
3	c.81–2 A > T				rs397509326	2	[22]
5	c.135–2 A > G		--		rs80358065	1	[27, 28]
5	c.211 A > G		p.Arg71Gly		rs80357382	3	[5]
7	c.303T > G		p.Tyr101Ter		rs80356936	1	[4, 29, 30]
8	c.442-1G > T		--		rs1351019392	1	[31]
11	c.959delG		p.Arg320AspfsTer20			1	Not described
11	c.1912delG		p.Glu638AsnfsTer13		rs80357933	1	[7–10]
11	c.1961delA		p.Lys654Serfs		rs80357522	1	[7–10]
11	c.3607 C > T		p.Arg1203Ter		rs62625308	1	[7–10]
11	c.4038_4039delAA		p.Gly1348AsnfsTer7		rs273900721	2	[7]
15	c.4591delG		p.Val1531fs		rs886040238	1	Not published
15	c.4675 + 1G > A		--		rs80358044	1	[33, 34]
18	c.5123 C > A		p.Ala1708Glu		rs28897696	8	[8]
20	c.5194-2A> G		--		rs80358069	1	[7, 11–13]
20	c.5266dupC		p.Gln1756fs		rs80357906	2	[26]
24	c.5536 C > T		p.Gln1846Ter		rs80356873	1	[35, 36]
	Large	Genomic		Rearrangements			
1ª-1B-2	*BRCA1*	Del Ex 1ª-1B-2				1	[14]
22 y 23	*BRCA1*	Del Ex 22 y 23		NM_007294.4		1	[14]

### Pathogenic variants of the BRCA2 gene

The detected pathogenic variants of the *BRCA2* gene are listed in Table 4. Among the *BRCA2*-positive families (*n* = 39), 24 different pathogenic variants were identified, including 8 recurrent pathogenic variants and 16 unique pathogenic variants.

The pathogenic variant c.6024dupG was detected in 6 unrelated families; c.2808_2011delACAA, in 5 families; and c.2457delA, c.2701delC, c.5116_5119delAATA, c.6275delTT, c.7558 C > T and c.7617 + 1G > A, in two families.

**Table 4:** Germline pathogenic variants of *BRCA2* in breast and ovarian cancer families in La Rioja

Exon	HGVS nomenclature at cDNA level	HGVS nomenclature at protein level	dbSNP	Number of families	References: articles published with the variant
3	c.262_263delCT	p.Leu88Alafs	rs276174825	1	[15, 16]
10	c.1128delT	p.Phe376LeufsTer23	rs80359263	1	[17, 18]
10	c.1363_1369delTCAGAGA	p.Glu456HisfsTer2	NM_000059.4	1	Not described
10	c.1397dupA	p.Asn466LysfsTer2	NM_000059.4	1	Not described
10	c.1586delTinsCA	p.Phe529SerfsTer2		1	[7]
11	c.2457delA	p.Asp820MetfsTer5	rs1322527832	2	[40]
11	c.2701delC	p.Ala902LeufsTer2	rs397507637	2	[4, 7, 8, 15, 17–21]
11	c.2808_2811delACAA	p.Lys936Glnfs	rs80359352	5	[4, 7–10, 15, 19, 38]
11	c.3264dupT	p.Gln1089SerfsTer10	rs80359380	1	[4, 7, 8, 15, 17–21]
11	c.3680_3681delTG	p.Leu1227GlnfsTer5	rs80359395	1	[17, 21]
11	c.3860delA	p.Asn1287Ilefs	rs80359406	1	[4, 7, 8, 15, 17–21]
11	c.4234_4236delACTinsC	p.Thr1412GlnfsTer10	NM_000059.4	1	Not described
11	c.5116_5119delAATA	p.Asn1706Leufs	rs276174853	2	[41, 42]
11	c.5576_5579delTTAA	p.Ile1859LysfsTer2	rs80359520	1	[4, 7, 8, 15, 17–21]
11	c.6024dupG	p.Gln2009AlafsTer8	rs80359554	6	[7, 37]
11	c.6275delTT	p.Leu2092ProfsTer7	rs11571658	2	[7, 8, 15, 43]
15	c.7558 C > T	p.Arg2520Ter	rs80358981	2	[4, 5]
15	c.7617 + 1G > A	--	rs397507922	2	[4, 18]
18	c.8021delA	p.Lys2674ArgfsTer2	rs397507952	1	[4]
19	c.8387delC	p.Pro2796LeufsTer25	NM_000059.4	1	Not described
20	c.8632 + 2T > G	--	rs397507998	1	[18]
23	c.9026_9030delATCAT	p.Tyr3009SerfsTer7	NM_000059.3	1	[8, 10, 15, 19–21,43]
24	c.9246dupG	p.Lys3083GlufsTer28	rs886038189	1	[45]
	Genomic	Rearrangements			
2	Del Ex 2			1	[14]

### Variants of unknown clinical significance

Thirteen variants of unknown significance (VUSs) were identified, four in *BRCA1* and nine in *BRCA2*. Table 5 shows the American College of Medical Genetics and Genomics (ACMG) classifications of the VUSs, as well as the allele frequencies found in the gnomAD database.

**Table 5:** *BRCA1* and *BRCA2* variants of unknown significance detected in families with breast and ovarian cancer

Gene	Exon	HGVS nomenclature	HGVS protein	ACMG classification	gnomAD exomes	gnomAD genomes
*BRCA1*	11	c.1661 A > G	p.Glu554Gly	Likely benign	No data	No data
	11	c.3047 A > G	p.Asn1016Ser	Likely benign	No data	No data
	11	c.4046 C > G	p.Thr1349Arg	Likely benign	No data	No data
	17	c.5017_5019delCAC	p.His1673delHis	Uncertain significance	No data	No data
*BRCA2*						
	7	c.632–3 C > G	(intron 7)	Pathologic	0.00000411	No data
		c.68-7del		Uncertain significance	No data	No data
	7	c.565G > T	p.Asp189Tyr	Uncertain significance	No data	No data
	10	c.1096T > G	p.Leu366Val	Likely benign	0.0000281	No data
	11	c.3170 A > G	p.Lys1057Arg	Uncertain significance	No data	No data
	11	c.3299 A > T	p.Asn1100Ile	Uncertain significance	0.0000131	No data
	11	c.3794G > A	p.Cys1265Tyr	Likely benign	No data	No data
	11	c.5729 A > T	p.Asn1910Ile	Likely benign	No data	0.0000318
	26	c.9530 A > G	p.Glu3177Gly	Uncertain significance	0.00000796	No data

### Study of families

The characteristics of the families are shown in supplementary Table 1. A total of 205 relatives in families with pathogenic variants were studied. Ninety-four individuals were heterozygous for pathogenic variants. Healthy heterozygous females were referred to the gynecology service for high-risk follow-up with surveillance and/or risk reduction surgery according to the patient’s age and pathogenic variant.

Family 37, with the pathogenic variant c.6024dupG in the *BRCA2* gene, included the greatest number of relatives studied, with 22 individuals. In Family 2, 12 relatives were studied, and 11 were positive.

Two relatives with breast cancer were studied in Families 12 and 32, one in each family, and no pathogenic variant was detected, a phenomenon known as phenocopy. In both patients, the complete study of both *BRCA1/2* genes was performed again, and the results were normal.

In Families 35, 51 and 53, the proband was a male with breast cancer. Pathogenic variants in the *BRCA2* gene (c.6024dupG, c.6275delTT and c.7558 C > T) were detected in all three patients.

## Discussion

Pathogenic variants in the *BRCA1/2* genes were detected in 16.5% of the study population. This diagnostic yield is lower than that in other published studies; however, we detected pathogenic variants in families that would not have been studied otherwise because they did not meet the inclusion criteria, as in the case of families without tumors. The population frequency of pathogenic variants has been estimated to be 1/400-1/800 [4]. Based on the population of La Rioja in 2019 and the number of subjects with pathogenic variants detected in our study, the population frequency of pathogenic variants was approximately 1/1920-1/1990, which is much lower than expected.

### Pathogenic variants of the BRCA1 gene

The pathogenic variant c.5123 C > A was detected in 8 unrelated families and was the most frequently described pathogenic variant in our population. The pathogenic variant c.211 A > G was detected in 3 unrelated families. This pathogenic variant is a founder pathogenic variant in Galicia, a region located in northwestern Spain [5], and one family in our study was of Galician origin (Family 9). The pathogenic variant c.81–2 A > T was found in 2 families and has not been described in the Spanish population. This variant destroys a canonical splice acceptor site and is predicted to cause abnormal gene splicing [22].

The pathogenic variant c.68_69delAG originated in Ashkenazi populations [23] and has one of the highest prevalence rates in Western European populations. Previous haplotype studies indicated a common origin of this pathogenic variant in Spanish (Sephardic Jews) and Ashkenazi Jewish (AJ) populations [24]. It is estimated that approximately 20% of the population of the Iberian Peninsula has Sephardic Jewish ancestry [25]. This pathogenic variant is widely described in the Spanish population; it is more common in Mediterranean areas and less common in the northwest-central zone. In our study, we found this variant in two families (Families 12 and 13). One of those families (Family 13) came from the city of Toledo, where an important Jewish population was located until the 15th century, and the other family came from the Mediterranean area. The female carrier of this pathogenic variant in Family 13 had gastric cancer and was assessed for pathogenic variants in other genes; after the variant in the *BRCA1* gene was identified, the patient underwent surveillance, and breast and ovarian cancer were detected.

The pathogenic variant c.5266dupC was originally described as a founder pathogenic variant in the AJ population. However, it is also present at appreciable frequencies in several European countries, raising questions about the origins of this pathogenic variant. The *BRCA1* c.5266dupC variant appears to have originated from a single common ancestor and was a common European pathogenic variant long before it became a founding AJ pathogenic variant; therefore, it is likely to be present in many other European countries [26]. In our study, it was found in two unrelated families. The patient in Family 31 was a Polish woman who did not know whether she had Ashkenazy ancestry; however, given her country of origin, we suspect this was possible. The other family (Family 30) was of Romanian origin.

The pathogenic variant c.135–2 A > G is not very frequent and has been described in Romanian and Pakistani populations [27, 28]. The patient in our study was of Romanian origin.

The pathogenic variant c.303T > C has not been described in the Spanish population, although it has been described in Chilean and Nigerian populations [4, 29, 30].

The c.442-1G > T variant is most likely pathogenic. It is located in the intronic region immediately upstream of exon 8 in *BRCA1* and causes abnormal splicing. However, this variant is of unknown origin in the ClinVar database. Experimental studies have shown that this variant disrupts the consensus splice site and potentiates a cryptic acceptor site in exon 7 (also known as exon 8 in the literature) located 3 nucleotides downstream of the natural splice site. This results in a deletion of 1 amino acid (p.Gln148del) but otherwise preserves the integrity of the reading frame [31]. This pathogenic variant was detected in an Ecuadorian family residing in La Rioja. Previous Latin American population studies did not include the Ecuadorian population [32].

The pathogenic variant c.959delG has not been described previously. This variant was detected in a healthy proband whose family included two breast cancer patients who were diagnosed at the ages of 33 and 47 years and had previously died.

The pathogenic variant c.4591delG has been described but not yet published. It was detected in a female with ovarian cancer diagnosed at age 37 years of Romanian origin.

The pathogenic variant c.4675 + 1G > A has been described in Western European and Brazilian populations but not in a Spanish population [33, 34].

The pathogenic variant c.5536 C > T was detected in a healthy female of Romanian origin (Family 30) whose relatives with cancer were deceased. It has been described previously in Canadian, Asian and Western European populations [35, 36].

Two large deletions were found, one in exon 1 A-1B2 and one in exons 22 and 23. Deletions spanning exons 22–23, referred to in the literature as exon 23–24 deletions, have been described in individuals and families affected by breast and/or ovarian cancer [14].

### Pathogenic variants of the BRCA2 gene

The pathogenic variant c.6024dupG was the most common variant in the *BRCA2* gene in our population and was detected in 6 unrelated families. Although it has not been widely described in the rest of Spain, it is common in Navarra, a region bordering La Rioja, and has been detected in 5 unrelated families [7]. De Sabando et al. [7] suggest that the c.6024dupG variant has a founder effect in southwestern Navarra. It has been described in one family each in several different Spanish-American countries, including Argentina, Mexico and Colombia, as well as in the Jewish population [4, 37]. Our study also supports this theory given the high number of families with this variant found in close geographical proximity, although complementary molecular studies would be necessary for confirmation. This article describes a high correlation of this variant with ovarian cancer. In our series, three of the 6 families with the variant had ovarian cancer (Families 33, 34, and 38), and in another family, the proband was a male with breast cancer (Family 35).

The pathogenic variant c.2808_2811delACAA has been described worldwide and in all Spanish regions with published data [4, 7–10, 15, 19, 38]. It is the most common variant in France and Colombia and in western and southern European countries. It is believed to have occurred approximately 80 (46–134) generations ago. Due to the diversity of the haplotypes, it is possible that they have independent origins [39].

The pathogenic variants c.1363_1369delTCAGAGA and c.1397dupA have not been described. These variants were detected in patients from Families 58 and 59, both of whom had breast and ovarian cancer and were from La Rioja.

The pathogenic variant c.1586delTinsCA has been described in Navarra, a neighboring community of La Rioja, but not in other areas of Spain or worldwide [7].

The pathogenic variant c.2457delA is described in the ClinVar database [40] but has not yet been published. In our study, we detected this variant in two families (Families 44 and 45) from La Rioja with several cases of breast cancer.

The pathogenic variant c.4234_4236delACTinsC has not been described. It was detected in a woman with pancreatic cancer whose brother had previously died from melanoma and therefore could not undergo genetic study (Family 64).

The pathogenic variant c.5116_5119delAATA has a foundational effect in the Spanish region of Castilla y Leon [41], although it has also been described in the Korean population [42]. In our study, this variant was detected in two unrelated families with several cases of breast cancer (Families 48 and 49).

The pathogenic variant c.6275delTT was found in two unrelated families (Families 50 and 51), one of which is a man with breast cancer. This variant has been described worldwide and in different regions of Spain [7, 8, 15, 43].

The pathogenic variant c.8021delA has been described worldwide [4] but not in the Spanish population. In our study, it was detected in a healthy female (Family 66) from Romania.

The pathogenic variant c.8387delC has not been described previously. The patient was a female with ovarian cancer (Family 67) from Ecuador.

The pathogenic variant c.9026_9030delATCAT, detected in one family, has been described in other regions of Spain [8, 10, 15, 19–21, 43]. A haplotype study of 12 families showed that all the families came from northeast Spain (Catalonia-Valencia) and shared a common haplotype, indicating a single origin. The results suggest that these families share a common ancestry with *BRCA2* c.9026_9030delATCAT, which is a founder pathogenic variant originating in northeast Spain, with an estimated age of 92 (95% CI 56–141) generations [44].

The pathogenic variant c.9246dupG has been described in the Colombian population, and the patient in our study was of Colombian nationality (Family 70). Cock-Rada et al. [45] reported this variant in five unrelated families, so this variant could have a foundational effect on the Colombian population.

### Variants of unknown clinical significance

The c.5017_5019delCAC (p.His1673del) variant in *BRCA1* has a contradictory interpretation of pathogenicity in the ClinVar database. Zuntini et al. [46] studied this variant in 14 unrelated families from the Emilia-Romagna region of Italy. In their study, they found that all probands shared the same rare haplotype at the *BRCA1* locus, suggesting a common ancestry in this region (northern Italy). Although the pathogenicity of the allele is strongly supported by a multifactorial relationship, the authors cannot exclude the possibility that p.His1673del is not deleterious per se but is linked to another undetected variant on the same allele. They also described a high frequency of ovarian cancer in relation to breast cancer at a ratio of 1:0.87 [46]. In the family studied in La Rioja, there were three cases of ovarian cancer, cosegregating with the variant studied with a high clinical suspicion of being pathogenic.

The c.632–3 C > G variant in *BRCA2* was classified as pathogenic by Houdayer et al. [40]. A study of messenger RNA was performed at our center to determine whether this change was due to a splicing error. After the RNA analysis, we were unable to demonstrate its pathogenicity. The patient with breast cancer was treated with olaparib and achieved a progression-free survival of 6 months with a partial response.

The c.3170 A > G variant in *BRCA2* was detected in a patient with the pathogenic variant c.7617 + 1G > A (Family 55). None of the three family members studied had either of these variants; therefore, we suspect that the VUS is processed on the same allele as the pathogenic variant. We think that c.3170 A > G could be a neutral variant and that c.7617 + 1G > A is the pathogenic variant.

### Spectrum of BRCA1/2 gene variants in La Rioja and that of other Spanish regions

The variants found in the La Rioja community were similar to those found in other communities in Spain. The pathogenic variant (PV) c.5123 C > A in the BRCA1 gene and the PV c2808_2811delACAA in the BRCA2 gene are present throughout Spain and are probably the most frequently found [8, 38].

The presence of emigrant populations causes the appearance of variants that were not present in the original Spanish population, such as the PV c.5266dupC in the BRCA1 gene, which could be found more frequently in one or two generations when they become permanently established in Spain. It also occurs in populations that have not had genetic studies carried out, as in the case of PV c.442-1G > T in BRCA1 of Ecuadorian origin, which had not been described until now.

The PV c.6424dupG, c.1586delTinsCA, and c.7558 C > T in BRCA2 are only described in the community of La Rioja and the community of Navarra, two neighboring regions in northern Spain, but not in other regions of Spain [7].

The PV c.211 A > G in BRCA1 originating from Galicia, the PV c.5116_5119delAATA in BRCA2 originating from Castilla y Leon, and PV c.9026_9030delATCAT in BRCA2 originating from Catalonia are also present in La Rioja, possibly due to population mobility in other regions of Spain [5, 41, 44].

The PV c.303T > C in BRCA1 has not yet been described in other regions of Spain. It has been found in only one family of Spanish origin. This variant has been described in other populations of Chilean and Nigerian origin, which we do not believe can be related to the family in our study [4, 29, 30].

The PV c.81–2 A > T in BRCA1 found in two families in our study has also not been described in other communities in Spain [4].

The PV c.68_69delAG in the BRCA1 gene is found more frequently in communities close to the Mediterranean such as Catalonia and Valencia in the eastern half of Spain and is not present in communities in the western half of Spain such as Galicia, Asturias, Castilla y Leon and the Basque Country [18, 19, 21].

As in other Spanish population studies, the number of PVs was slightly higher in the BRCA2 gene than in the BRCA1 gene, and males with breast cancer in our study only had PVs in BRCA2 [7, 8, 10].

## Conclusions

The spectrum of *BRCA1/2* pathogenic variants in La Rioja is similar to that in other Spanish regions, with some unique features. The pathogenic *BRCA2* variant c.6024dupG could have a founder effect in the Ebro riverbank area of the La Rioja and Navarra regions. Our study supports genetic testing in healthy individuals belonging to families with a high cancer risk, as we found pathogenic variants in 20% of these individuals. In Group I-D (single case of triple-negative breast cancer diagnosed ≤ 50 years old), we detected a high probability of finding pathogenic variants in *BRCA1/2* genes. We describe for the first time the pathogenic variants c.959delG in *BRCA1* and c.1363_1369delTCAGAGA, c.1397dupA, c.4234_4236delACTinsC and c.8387delC in *BRCA2*.

## Electronic supplementary material

Below is the link to the electronic supplementary material.

Supplementary Material 1

## Data Availability

The datasets used and/ or analysed during the current study are available from the corresponding author on reasonable request.

## Abbreviations

BIC: Breast Cancer Mutation Database
SEOM: Spanish Society of Clinical Oncology
NGS: Next-generation sequencing
VUS: Variant of unknown clinical significance
MLPA: Multiplex ligation-dependent probe amplification
HGMD: Human Gene Mutation Database
PCR: Polymerase chain reaction
EDTA: Ethylenediaminetetraacetic acid
CNV: Copy number variant
AJ: Ashkenazi Jewish
